# Yeasts of the soil – obscure but precious

**DOI:** 10.1002/yea.3310

**Published:** 2018-03-02

**Authors:** Andrey M. Yurkov

**Affiliations:** ^1^ Leibniz Institute DSMZ‐German Collection of Microorganisms and Cell Cultures Inhoffenstraße 7B 38124 Brunswick Germany

**Keywords:** agriculture, biogeography, biotechnology, endemism, forest, taxonomy, traits

## Abstract

Pioneering studies performed in the nineteenth century demonstrated that yeasts are present in below‐ground sources. Soils were regarded more as a reservoir for yeasts that reside in habitats above it. Later studies showed that yeast communities in soils are taxonomically diverse and different from those above‐ground. Soil yeasts possess extraordinary adaptations that allow them to survive in a wide range of environmental conditions. A few species are promising sources of yeast oils and have been used in agriculture as potential antagonists of soil‐borne plant pathogens or as plant growth promoters. Yeasts have been studied mainly in managed soils such as vineyards, orchards and agricultural fields, and to a lesser extent under forests and grasslands. Our knowledge of soil yeasts is further biased towards temperate and boreal forests, whereas data from Africa, the Americas and Asia are scarce. Although soil yeast communities are often species‐poor in a single sample, they are more diverse on the biotope level. Soil yeasts display pronounced endemism along with a surprisingly high proportion of currently unidentified species. However, like other soil inhabitants, yeasts are threatened by habitat alterations owing to anthropogenic activities such as agriculture, deforestation and urbanization. In view of the rapid decline of many natural habitats, the study of soil yeasts in undisturbed or low‐managed biotopes is extremely valuable. The purpose of this review is to encourage researchers, both biologists and soil scientists, to include soil yeasts in future studies.

## 1 HISTORY

**Figure 1:**
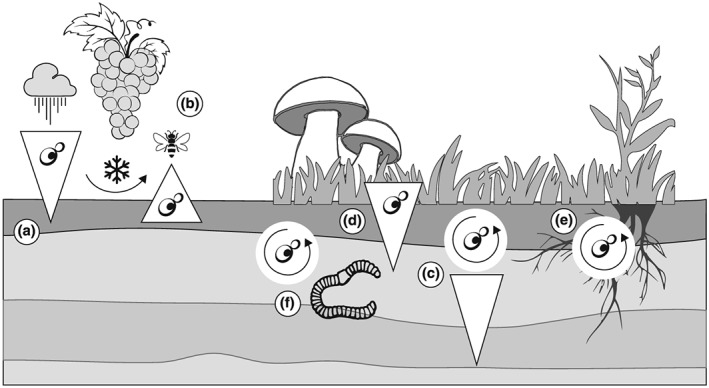
Schematic representation of ecology and dispersal routes of yeasts in soils. Yeasts from ripe fruits are carried to the soil (a), hibernate during the winter and inoculate fruits above‐ground (b). Indigenous soil yeasts multiply in the topsoil, and their number decreases in deeper soil layers (c). The presence of some yeasts is related to the deposition of plant and animal residues on the soil; these transient species are quickly eliminated (outcompeted or preyed on) in the soil (d). Some yeasts are associated with soil plant toots (e) and invertebrates (f)

In the years following the first observation of yeasts in 1680 by Antonie van Leeuwenhoek, these small eukaryotic organisms were considered to be associated mainly with alcoholic fermentation of beer and wine. However, the question of the origin of yeasts found in fermented products soon became the starting point for research on yeasts outside man‐made environments. Louis Pasteur was one of the first to attempt to answer this question. In 1875, he began a series of investigations to find out whether yeasts could be isolated from the skin of the grapes used in making wine and whether they were present only at one time of the year (reviewed in Guilliermond, 1920). His experiments indicated that, during autumn, yeasts existed on practically all parts of the vine and disappeared during the winter. Emil Hansen investigated the life cycle of the yeast *Saccharomyces apiculatus* (Hanseniaspora uvarum) that was widespread on fruits (Hansen, 1880). He thought that yeasts were distributed by air currents, insects and rainfall to other fruits as well as to the soil on which fruit trees grow (reviewed in Guilliermond, 1920; Starkey & Henrici, 1927; Bouthilet, 1951). Hansen was also able to observe living yeasts in soil under fruit trees. Using both cultivation and artificial inoculation experiments, he demonstrated that yeasts can survive in soils throughout the year. Yeasts have been frequently observed in the surface layers but rarely in the deeper soil layers (Figure 1a). In the following years yeasts were found in soils of vineyards and orchards down to a depth of 12–13 and 20–30 cm by the pioneering microbiologists Amedeo Berlese and Hermann Müller‐Thurgau, respectively (reviewed in Starkey & Henrici, 1927). Hansen believed that yeasts hibernating in soil during the winter were carried by the wind on dust particles and inoculated fruits above‐ground (reviewed in Guilliermond, 1920; Starkey & Henrici, 1927), while Berlese suggested that insects served as vectors of yeast cells (Figure 1b) (reviewed by Brysch‐Herzberg, 2004). Later, Hansen investigated the presence of yeasts in soils in the Copenhagen area and also found them outside orchards and gardens under beech, fir, pine and oak trees, although only in about 30% of samples (reviewed in Guilliermond, 1920).

Other pioneering studies demonstrated that yeasts are present in soils (reviewed in Guilliermond, 1920; Starkey & Henrici, 1927; Bouthilet, 1951). However, they were not recognized as indigenous soil organisms and the ability of yeasts to propagate in soils was repeatedly questioned (discussed by Danielson & Jurgensen, 1973; Phaff, Miller, & Mrak, 1966; Phaff & Starmer, 1987). Yeasts were often equated with fermenting ascomycetes that colonize above‐ground sugar‐rich substrates, such as ripe fruits and flowers. Starkey and Henrici (1927) and Ciferri (1928) analysed yeast numbers and noticed that bacteria and filamentous fungi outnumber yeasts in most soils. The low quantity of yeasts was interpreted as evidence for the minor importance of this group of fungi in soil functioning (e.g. Starkey & Henrici, 1927 and in later reviews by Phaff & Starmer, 1987; Starmer & Lachance, 2011). Starkey and Henrici (1927) did not find any correlation between the occurrence of yeasts and the type of soil, vegetation or season of the year. In contrast, Pumpyanskaya (1938) showed that yeast quantities depend on physical and chemical soil properties (reviewed in Babjeva & Golovleva, 1963). It is important to mention that approaches that were used at that time to study yeasts strongly favoured the isolation of fast‐growing fermenting ascomycetes. Margret di Menna (1957) revised isolation techniques used for soil yeasts. She showed that suitable culture media, cultivation conditions and sample pre‐treatments resulted in higher yeast colony counts. Culture media, supplements and incubation techniques have been changing with the evolving knowledge of the taxonomic composition of soil yeast communities and the ecology of the dominant species (e.g. Babjeva, 1969; Boundy‐Mills, 2006; di Menna, 1959; Miller & Webb, 1954). The use of nitrogen‐free media (e.g. Brown's Azotobacter agar) facilitated the isolation of slow‐growing *Lipomyces* species from soils. Reliable isolation techniques and a growing number of studies showed that yeast numbers in soils may exceed those on decaying plant material and convinced researchers that yeasts do live, and not only reside, in soils. A number of soil‐borne yeasts were isolated and described during the beginning of the twentieth century, e.g. *Apiotrichum dulcitum*, *A. porosum*, *Cyberlindnera saturnus* (originally *Willia saturnus*), *Lipomyces starkeyi*, *Nadsonia starkeyi‐henricii* (originally *Schizoblastosporion starkeyi‐henricii*), *Schwanniomyces polymorphus* (originally *Pichia polymorpha*) and *Vanrija humicola* (originally *Torula humicola*). As outlined by Phaff and Starmer (1987), the repeated isolation of the same yeasts from soils and their absence in other sources above‐ground were employed as another argument to demonstrate the soil origin of several yeast species.

A great variety of yeast species have been isolated from soils (Vadkertiová, Dudášová, & Balaščáková, 2017; Yurkov, 2017), but only starting in the 1950s–1960s did yeast surveys become quantitative. The diversity of soils and vegetation types encouraged scientists to study yeasts across different climates, biotopes and soil types. Di Menna (1960, 1965a) was probably the first scientist to study soil yeasts through a series of biotopes characterized by different types of vegetation, land management and soil properties. Jensen (1963) analysed yeasts in Danish beech forests in different seasons. Capriotti (1967) sampled soils across a wide geographical range in the USA. Starting in 1956, Johannes van der Walt described more than 30 yeast species from soils, many of which were isolated in South Africa (Smith & Groenewald, 2012). Later, in cooperation with Maudy Smith, he intensified studies of the yeast family Lipomycetaceae from an evolutionary perspective. Inna Babjeva and co‐workers systematically studied the distribution of yeasts in major soil types in the USSR. Babjeva and Golovleva (1963) provided the first comprehensive review of soil yeasts in zonal, intrazonal and azonal soils (see also Babjeva & Chernov, 1995). Helen Vishniac analysed soils collected over a period of nearly 30 years along a latitudinal gradient in western North America covering polar to tropical climates (Vishniac, 2006a). At about the same time, Ivan Chernov (2005) performed a similar study. He collated data derived from a total of 114 localities and ca. 7000 samples previously analysed by Babjeva and co‐workers in order to study the influence of geographic latitude and natural zones on yeast community parameters. Research on interactions of soil yeasts with vegetation has intensified during the last two decades. In particular, studies performed by Renata Vadkertiová and co‐workers and Chernov and co‐workers address the influence of tree species (e.g. Sláviková & Vadkertiová, 2000; Maksimova & Chernov, 2004; Yurkov, Inácio, Chernov, & Fonseca, 2015), land management (Sláviková & Vadkertiová, 2003), invasive plants (Glushakova, Kachalkin, & Chernov, 2015b) and temporal changes (Golubtsova, Glushakova, & Chernov, 2007; Sláviková & Vadkertiová, 2000).

Soil yeasts have been included in book chapters covering major advances in yeast ecology, e.g. do Carmo‐Sousa (1969), Phaff, Miller, and Mrak (1978), Phaff and Starmer (1987), Spencer and Spencer (1997) and Starmer and Lachance (2011). Babjeva and Gorin (1987) provided a comprehensive review of the past research on *Lipomyces* species. Recently, Alfred Botha wrote two reviews solely dedicated to soil yeasts (Botha, 2006, 2011). These sources and the two recent book chapters by Vadkertiová et al. (2017) and Yurkov (2017) are recommended.

**Future research on soil yeasts**

The distribution of soil yeasts is patchy, uneven and not always influenced by soil abiotic parameters. What factors shapes the community? Do yeasts rely on soils properties or do they depend on natural hosts and vectors? Where do soil yeasts actually come from?

Many soil yeasts are polytrophic species that are able grow on diverse carbon and nitrogen sources. Does this mean that they are functionally redundant in soils? What is the basis for redundancy? Is phylogenetic diversity a better marker than functional traits? Do similar physiologies mean redundancy?

Distribution patterns appear not at the species level, but at the level of higher taxonomic ranks. Some yeast genera are found frequently, but not always exclusively, in soils, i.e. *Apiotrichum*, *Barnettozyma*, *Lipomyces*, *Saitozyma*, *Schwanniomyces*, and *Solicoccozyma*. Does this reflect an evolutionary adaptation of these genera to soils? Basidiomycetous yeasts are usually more abundant in soils although ascomycetes may outnumber them in managed soils. Why?

Yeasts could be an important source of carbon in soil. Yeast species frequently encountered in soil often utilize hemicellulose‐derived sugars and intermediates of lignin degradation. What is the role of yeasts in the soil food web? What is the link to litter and deadwood decomposition? What organisms depend on yeasts as carbon and nitrogen sources? How do yeasts interact with other soil organisms?

More thorough and systematic sampling of soil worldwide is needed outside of temperate and boreal zones, generally from Asia, Africa and the American continent, and from unmanaged soils, particularly in the tropics and the subtropics.

## 2 PROPERTIES OF SOILS AND DIVERSITY OF SOIL TYPES

The soil cover of the Earth is diverse in terms of its mineral composition, organic matter characteristics, soil‐forming processes, climate and management. Vasily Dokuchaev was the first to link soil hydrological and geochemical conditions to the history of the above‐ground vegetation and climate (reviewed by Fairbridge, 2008). He also developed the first soil classification scheme based on the combination of climatic, abiotic and biotic factors responsible for soil formation. His contribution is broadly acknowledged in soil science as all existing soil classification systems rely on both properties and processes. Soil formation processes encompass chemical and physical factors that change organic and inorganic fractions and, thus, predict the range of the most relevant parameters. Additionally, soil processes reflect the history of the habitat and the factors that shaped yeast communities in the past. Thus, a few easily determined basic soil properties in the field (e.g. temperature, pH, conductivity and soil texture) would potentially provide less information than the identification of the soil type. A common effort has been made by soil scientists to unify existing soil classifications into a single system presently known as the World Reference Base for Soil Resources (IUSS Working Group WRB, 2014). With these recent guidelines in hand, soil types can be determined in the field according to the existing national resources (e.g. soil maps) and then translated into a common system that will be understood by scientists worldwide.

Many studies conducted in the past have focused on the description of new yeasts and did not always provide information on other yeasts isolated from the same soils, such as the valuable taxonomic works by Capriotti, Phaff, van der Walt and Wickerham. Our knowledge of soil yeasts is biased towards temperate and boreal climates. Soils in the former USSR have been intensively surveyed by Babjeva, Chernov and co‐workers (e.g. Babjeva & Chernov, 1995; Chernov, 2005). Forest and grassland soils in Europe were studied in Austria, Czech Republic, Denmark, Italy, Germany and Slovakia (e.g. Jensen, 1963; Mašínová et al., 2017; Sláviková & Vadkertiová, 2000, 2003; Wuczkowski & Prillinger, 2004; Yurkov, Kemler, & Begerow, 2012). Cold Arctic and Antarctic soils have been fairly well investigated during the past six decades (reviewed by Vishniac, 2006a, 2006b; Connell, Rodriguez, Redman, & Dalluge, 2014; Zalar & Gunde‐Cimerman, 2014). In contrast, data from temperate and tropical soils in Asia, Africa and both Americas are scarce (e.g. Mok, Luizao, da Silva, Teixeira, & Muniz, 1984; Spencer & Spencer, 1997; Takashima et al., 2012; Vishniac, 2006a). It is important to point out that tropical biotopes (e.g. rain forests) have received least attention despite their importance as major biodiversity hotspots. For example, both large studies of yeasts from Amazon rain forests in Brazil had an applied focus and surveyed either species pathogenic to humans (Mok et al., 1984) or yeasts producing killer toxins (Vital, Abranches, Hagler, & Mendonça‐Hagler, 2002). Similarly, Asian soils were mainly studied as the source of novel yeast species but the information on the distribution of other species is scarce (e.g. Jaiboon, Lertwattanasakul, Limtong, & Limtong, 2016; Landell et al., 2014; Limtong, Yongmanitchai, Kawasaki, & Fujiyama, 2009; Limtong, Yongmanitchai, Tun, Kawasaki, & Seki, 2007). Forest soils in the Southern Hemisphere are strongly under‐sampled. The temperate silver beech (*Nothofagus pumilo*) forest was studied in Patagonia (Mestre, Rosa, Safar, Libkind, & Fontenla, 2011). Decaying wood and, to a lesser degree, soil in the Valdivian temperate rain forest in Chile were extensively sampled in the past by Grinbergs, González and Ramírez (reviewed in Phaff & Starmer, 1987; González, Martínez, Almendros, & Grinbergs, 1989). Di Menna (1965a) performed a broad survey of New Zealand soils. However, very few cultures isolated during this study were retained, with the result that the majority of yeast names used at that time cannot be confidently translated into the currently used nomenclature. It is generally difficult to evaluate the results obtained before 1970s owing to obsolete identification approaches and the lack of original cultures for re‐identification. Because biological diversity is often seen as a natural resource or capital of a country, its exploration, including biodiversity assessments, is seriously hampered by the restrictions resulting from international and national regulations that originally aimed to protect the local biodiversity (Boundy‐Mills et al., 2016; Overmann & Scholz, 2017). As a result, the strict control of the access to biological resources, both biotopes and organisms, complicates the work of local researchers and discourages international collaborators from conducting research projects in developing countries, where the majority of biodiversity hotspots are located (Overmann & Scholz, 2017).

## 3 SOIL YEAST COMMUNITIES: DIVERSITY AND TAXONOMY

Yeasts have been recovered from various soil types, including extreme acid, alkaline, volcanic and cryogenic soils. Unlike in above‐ground sources, soil yeasts are not numerous; their numbers rarely exceed thousands of cells per gram, although counts reaching millions of cells are occasionally reported (Botha, 2006; Phaff & Starmer, 1987). Soils rich with organic matter usually yield higher yeast colony numbers (e.g. Botha, 2006); yeast abundances are higher in fertilized agricultural soils (Vadkertiová et al., 2017) and in non‐fertilized temperate and boreal soils, where organic matter decomposition rates are slow (e.g. Babjeva & Chernov, 1995; Babjeva & Golovleva, 1963; Chernov, 2005). However, subtropical and tropical soils are insufficiently sampled to allow any well‐supported generalizations to be drawn. The quantity of yeast cells usually decreases with soil depth (Figure 1c), a trend that has been explained by the amount of available nutrients and soil organic matter (e.g. Botha, 2006; Danielson & Jurgensen, 1973; Maksimova & Chernov, 2004; Starmer & Lachance, 2011). Viable yeasts (*Lipomyces tetrasporus*) were observed in soil layers as deep as 100 cm (Vinovarova & Babjeva, 1987) and even 200 cm (Glushakova, Kachalkin, Tiunov, & Chernov, 2017), although soil yeasts become exceedingly rare below the top 20–30 cm (e.g. Maksimova & Chernov, 2004; Phaff & Starmer, 1987; Wuczkowski & Prillinger, 2004). Yeasts have been found in bulk soil (Figure 1c), rhizosphere (Figure 1e) and in association with invertebrates (Figure 1f).

Although soil communities are frequently regarded as species poor, low species richness in a single plot (alpha diversity) contrasts with the larger number of yeasts that can be isolated from a forest or a region. The distribution of yeasts in soils is often fragmented with a few species only shared between sampling sites. For example, Vishniac (2006a) reported nearly 40% of yeasts to be restricted to a single locality. Likewise, temperate forests in Germany (three regions) had only *Apiotrichum dulcitum* in common (Yurkov et al., 2012). Three Mediterranean xerophyl forests sampled in a single locality had eight out of 57 species shared between all three plots (Yurkov, Röhl, et al., 2016). The dissimilarity in species composition between sites results in high diversity values on the regional level (e.g. Yurkov, Kemler, & Begerow, 2011; Yurkov, Röhl, et al., 2016). Recent studies showed that fairly well analysed soils yield a large number of as yet undescribed yeasts. The proportion of potentially novel taxa was estimated to exceed 30% in temperate beech and Mediterranean xerophyll forests (Yurkov, Röhl, et al., 2016; Yurkov, Wehde, et al., 2016). The same holds true for a few other temperate forests (Mašínová et al., 2017; Mestre et al., 2011; Takashima et al., 2012) and is likely to be true for tropical biotopes.

Not every yeast species isolated from soil is an indigenous soil inhabitant but may originate from other sources other than soils (e.g. Phaff et al., 1978; Phaff & Starmer, 1987). For example, pigmented *Cystobasidium*, *Rhodotorula*, *Rhodosporidiobolus*, *Sporobolomyces* and *Vishniacozyma* species from plant surfaces were frequently recovered from soils (Figure 1d). Species of the Basidiomycete genera *Cystofilobasidium* and *Apiotrichum* as well as non‐pigmented Microbotryomycetes (e.g. *Bannozyma*, *Colacogloea*, *Curvibasidium*, *Hamamotoa* and *Oberwinklerozyma*) are shared sometimes between topsoil and forest litter layers (reviewed by Yurkov, 2017). Observation of fermenting ascomycetous yeasts frequently found on fruit surfaces, such as *Hanseniaspora* species, suggests that they reside in soils (e.g. Phaff & Starmer, 1987). However, the ability to ferment sugars does not predict well the transient habit of a yeast species, since several autochthonous soil yeasts possess this trait, e.g. *Barnettozyma*, *Cyberlindnera*, *Kazachstania* and *Schwanniomyces*. Ascomycetous yeasts are generally more frequent and abundant in agricultural soils, orchards and grasslands (Vadkertiová et al., 2017; Yurkov et al., 2012). Ascomycetous yeasts of the genus *Lipomyces* are typical soil yeasts, some of which (*L. starkeyi* and *L. tetrasporus*) are distributed worldwide (Kurtzman & Smith, 2011). The genus *Myxozyma* represents asexual forms of *Lipomyces*. Interestingly, several *Myxozyma* and *Lipomyces* species have been isolated from insect‐associated habitats such as frass, decaying cactus tissues and tree fluxes (Kurtzman & Smith, 2011).

Basidiomycetes are dominant in forest soils, and yeasts of the former polyphyletic genus *Cryptococcus* are among the most frequently reported soil species (e.g. Babjeva & Chernov, 1995; Botha, 2006; Vishniac, 2006a; Yurkov et al., 2012). Yeasts of the genera *Cryptococcus* and *Trichosporon* were also reported among the most numerous fungal operational taxonomic units (OTU as a proxy for species) in culture‐independent surveys (reviewed in Yurkov, 2017). However, the problem of erroneous species naming or improper taxonomic assignment hampers identification of yeasts in culture‐independent surveys. Molecular OTUs that are often reported as members of large polyphyletic phenotypic genera and not assigned to the particular phylogenetic lineage or clade provide no or limited ecological information (discussed in Yurkov, 2017). The ongoing reclassification of yeasts in polyphyletic genera *Cryptococcus*, *Rhodotorula* and *Trichosporon* is believed to ease the communication of results by distinguishing yeast species from related phylogenetic lineages or clades. As a result of the sequence‐based reclassification of the genus *Cryptococcus* (Liu et al., 2015), soil‐related species have been accommodated in the following genera: *Goffeauzyma*, *Heterocephalacria*, *Hannaella*, *Holtermanniella*, *Naganishia*, *Papiliotrema*, *Piskurozyma*, *Saitozyma*, *Solicoccozyma* and *Vanrija* (Table 1). *Trichosporon* is another prominent yeast genus reported from soils. This genus has been recently reclassified (Liu et al., 2015) and common soil‐related species have been transferred in the genera *Apiotrichum*, *Cutaneotrichosporon* and *Tausonia* (Table 1). Older studies reported the species Cryptococcus albidus, *Cryptococcus curvatus*, *Cryptococcus humicola*, Cryptococcus laurentii and *Trichosporon cutaneum* (also as *Trichosporon beigelii*) from soils on the basis of growth characteristics. However, re‐identification of these yeast cultures with DNA‐based tools has been performed in only a few cases. While *Naganishia albida* and *Tausonia pullulans* were repeatedly identified in soils (although not as dominating species), the clinically relevant *Trichosporon cutaneum* does not inhabit soils. Although the isolation of *Saccharomyces* species from soils has been reported in the literature (e.g. Brysch‐Herzberg & Seidel, 2017; Kowallik & Greig, 2016; Sampaio & Gonçalves, 2008; Sampaio & Gonçalves, 2017; Sniegowski, Dombrowski, & Fingerman, 2002; Sylvester et al., 2015), this yeast should be viewed as a transient soil species propagating on above‐ground substrates (e.g. fruits, bark, leaves, tree fluxes) and residing in soils (Sampaio & Gonçalves, 2017). In most cases the isolation of these yeasts have been made from soils using a sugar‐rich enrichment culturing medium, with or without 7–8% (v/v) ethanol (Kowallik & Greig, 2016; Sampaio & Gonçalves, 2008; Sniegowski et al., 2002; Sylvester et al., 2015). Such selective conditions allow the isolation of *Saccharomyces* but not most of indigenous soil yeasts. Reports of these yeasts outside vineyard and orchard soils are extremely rare and most of them have been made from broadleaf (oak, beech, southern beech) forest litter and the underlying topsoil (Kowallik & Greig, 2016; Mestre et al., 2011; Sampaio & Gonçalves, 2008; Sylvester et al., 2015).

**Table 1:** Reclassification of Tremellomycetes frequently isolated from soils

Order and family	Genus	Selected species
Tremellales		
Bulleribasidiacea	*Hannaella*	Cryptococcus luteolus
Rhynchogastremaceae	*Papiliotrema*	Cryptococcus laurentii *, Cryptococcus terrestris*
Trimorphomycetacea	*Saitozyma*	*Cryptococcus podzolicus*
Trichosporonales		
Trichosporonaceae	*Cutaneotrichosporon*	*Cryptococcus curvatus, Trichosporon moniliiforme*
	*Apiotrichum*	*Trichosporon dulcitum, Trichosporon laibachii, Trichosporon lignicola, Trichosporon loubieri, Trichosporon porosum*
	*Vanrija*	*Cryptococcus humicola*
Holtermanniales	*Holtermanniella*	*Cryptococcus watticus*
Filobasidiales		
Filobasidiacea	*Naganishia*	Cryptococcus albidus
	*Heterocephalactria*	*Cryptococcus arrabidensis*
	*Filobasidium*	
	*Goffeauzyma*	*Cryptococcus gastricus, Cryptococcus gilvescens*
Piskurozymaceae	*Solicoccozyma*	*Cryptococcus aerius, Cryptococcus terricola,* Cryptococcus terreus
	*Piskurozyma*	*Cryptococcus cylindricus*
Cystofilobasidiales		
Mrakiacea	*Krasilnikovozyma*	*Cryptococcus huempii*
	*Tausonia*	*Trichosporon pullulans*

## 4 YEAST PHENOTYPES

The presence of fermenting yeasts below‐ground was traditionally viewed as the result of contamination from above‐ground sources. However, species of the genera *Barnettozyma* (formerly *Williopsis* and *Zygowilliopsis*), *Cyberlindnera* (formerly *Pichia* and *Williopsis*), *Kazachstania* (formerly *Arxula* and *Saccharomyces*) and allied *Candida* species are prominent in grassland and agricultural soils (reviewed in Vadkertiová et al., 2017). These yeasts display a typical copiotrophic lifestyle; they grow fast, consuming simple sugars but not complex substrates, and are capable of anaerobic fermentation. The importance of fermentation in soil has not been investigated. However, the ability to utilize sugars in the absence of oxygen (e.g. when soil pores are filled with water) is potentially useful for soil yeasts.

Unlike the typical saccharolytic phenotype often attributed to yeasts, basidiomycetous species are able to utilize a wide spectrum of carbon sources, including complex compounds (e.g. Fonseca, 1992; Middelhoven, 1993; Sampaio, 1999). In his review on soil yeasts, Botha (2006) noted that most of the yeast species frequently encountered in soil are able to utilize the hemicellulose‐derived sugars l‐arabinose, d‐xylose and cellobiose (see also di Menna, 1959; Mestre et al., 2011; Sláviková & Vadkertiová, 2000). Some of the frequently encountered yeasts in soil were also found to assimilate intermediates of lignin degradation i.e. ferulic, 4‐hydroxybenzoic and vanillic acids (e.g. Botha, 2006; Henderson, 1961; Yurkov, Röhl, et al., 2016).

Species frequently found in soil are able to grow in media with low concentrations of nutrients (Babjeva & Gorin, 1987; Kimura et al., 1998; Vishniac, 1983). In particular, nitrogen oligotrophy is a widespread adaptation of yeasts, which enables them to colonize diverse substrates such as plant surfaces (reviewed by Fonseca & Inácio, 2006), tree fluxes (Golubev, Babjeva, & Novik, 1977) and soils (Botha, 2011). This adaptation is important because most soil nitrogen (some 96–98%) is bound within organic matter as complex insoluble polymers such as chitin, proteins and nucleic acids (van der Heijden, Bardgett, & Van Straalen, 2008). Interestingly, typical soil yeasts from the genus *Lipomyces* have the ability to assimilate nitrogen incorporated into heterocyclic compounds, such as imidazole, pyrimidine and pyrazine (LaRue & Spencer, 1968; van der Walt, 1992; Cornelissen, Botha, Conradie, & Wolfaardt, 2003). Recent studies showed that the diversity of yeasts growing on imidazole is larger and includes both asco‐ and basidiomycetes (Cornelissen et al., 2003; Yurkov et al., 2011; Yurkov, Wehde, et al., 2016). However, unlike typical oligotrophic organisms, many yeast species are able to grow in a wide range of nutrient concentrations on dilute and nutrient‐rich media (e.g. di Menna, 1957; Yurkov et al., 2011).

The other adaptation frequently reported to be advantageous for soil microorganisms is the ability to produce extracellular polysaccharide capsules (EPS). The formation of capsules is a known mechanism that enables microbes to sequester and concentrate nutrients while growing in low‐nutrient environments or sustain low water activity and desiccation (Aksenov, Babjeva, & Golubev, 1973; di Menna, 1959; Raspor & Zupan, 2006). Semi‐arid soils, low in nutrients and moisture, are mostly populated by encapsulated anamorphic basidiomycetous yeasts (Spencer & Spencer, 1997; Vishniac, 2006a). The ability of some of these soil yeasts to survive in sandy soils owing to the production of EPS has been demonstrated with the soil yeast *Naganishia albida* (formerly Cryptococcus albidus, Vishniac, 1995). Soil‐borne *Naganishia* and *Solicoccozyma* species (Cryptococcus diffluens and Cryptococcus terreus; di Menna, 1959) were viable after storage for 9 months in the dry stage. Yeast EPS and cell hydrophobicity (some *Apiotrichum* species; personal observation) impacts the adhesion, stability of biofilms and access to nutrients (e.g. Davey & O'Toole, 2000; Raspor & Zupan, 2006).

## 5 BIOTECHNOLOGICAL AND CLINICAL RELEVANCE

In his reviews on soil yeasts, Botha (2006, 2011) provided a detailed overview of the importance of yeasts for soil‐related processes, including nutrient transformations and maintenance of soil structure. Yeast EPS improves the stability of soil aggregates, affecting water‐holding capacity and soil fertility (Botha, 2011). Capsules also provide a habitat for associated soil bacteria, including nitrogen‐fixing bacteria (Babjeva & Gorin, 1987; Cojho, Reis, Schenberg, & Döbereiner, 1993; Dommergues & Mutaftschien, 1965; Metcalfe & Chayen, 1954). Soil yeasts solubilize macronutrients such, as P and Ca, making them available for plants (e.g. Fu et al., 2016; Mestre, Fontenla, Bruzone, Fernández, & Dames, 2016).

Indole‐3‐acetic acid (IAA), an auxin, is the most common phytohormone occurring in plants. It has great importance for plant growth and development processes, most often in combination with other phytohormones such as cytokinin or gibberellin. Reports available to date suggest that IAA synthesis is a frequent trait among yeasts (e.g. El‐Tarabily & Sivasithamparam, 2006; Limtong & Koowadjanakul, 2012; Streletskii, Kachalkin, Glushakova, Demin, & Chernov, 2016). Among autochthonous soil yeasts the following species have been studied for IAA synthesis: *Goffeauzyma gastrica*, *Holtermanniella takashiame*, *Papiliotrema laurentii*, *Piskurozyma cylindrica*, *Saitozyma podzolica*, *Solicoccozyma terrea*, *Solicoccozyma terricola*, *Tausonia pullulans* and *Vanrija albida* (Limtong, Kaewwichian, Yongmanitchai, & Kawasaki, 2014; Mestre et al., 2016; Streletskii et al., 2016). High amounts of IAA (>1000 μg/g) have been detected in *Saitozyma podzolica* and *Solicoccozyma terricola* (Streletskii et al., 2016 and references therein). However, the production of plant growth promoting compounds is often evaluated *in vitro* without testing the effects under glasshouse and field conditions (reviewed in El‐Tarabily & Sivasithamparam, 2006). Field studies are rare. IAA producing soil‐borne yeast *Cyberlindnera* (formerly *Williopsis*) *saturnus* enhanced the growth of maize plants (Nassar, El‐Tarabily, & Sivasithamparam, 2005).

Soil yeasts inhabit and interact with the plant rhizosphere (Figure 1f; Botha, 2011; Mestre et al., 2011). They were also studied as potential antagonists of soil‐borne plant pathogens (reviewed in El‐Tarabily & Sivasithamparam, 2006; Botha, 2011). Several yeast cultures originating from the rhizosphere were reported to reduce rates of plant diseases (reviewed in Botha, 2011; Fu et al., 2016). Different species of yeasts also showed different mechanisms of antagonism towards growth of fungal root pathogens (e.g. Botha, 2011; El‐Tarabily & Sivasithamparam, 2006). However, only a few of the tested potential biocontrol species are true soil inhabitants. *Barnettozyma californica* and *Galactomyces candidum* isolated from the rhizosphere of *Drosera spatulata* exhibited significant antagonistic effects against *Glomerella cingulata* in culture (Fu et al., 2016). Likewise, the soil yeast *Vanrija albida* showed the best negative effect on the growth of plant pathogens *Verticillium dahliae* and Pythium aphanidermatum (Mestre et al., 2016).

Oleaginous yeasts are promising agents for biofuel production (e.g. Ageitos, Vallejo, Veiga‐Crespo, & Villa, 2011; Passoth, 2017; Sitepu et al., 2014). Among them several soil‐borne yeast genera have been studied, including *Apiotrichum* (formerly *Trichosporon porosum*), *Cutaneotrichosporon* (formerly *Cryptococcus curvatus*), *Lipomyces*, *Saitozyma* (formerly *Cryptococcus podzolicus*) and *Solicoccozyma* (formerly *Cryptococcus terricola*) (e.g. Pan et al., 2009; Schulze et al., 2014; Sitepu et al., 2014; Tanimura et al., 2014).

Pathogenic yeasts Cryptococcus neoformans, *Coccidioides immitis*, several clinically relevant *Candida* and species formerly classified in the genus *Trichosporon* can be found in soils (e.g. Miceli, Díaz, & Lee, 2011). However, the proportion of yeasts from rural soils growing at elevated temperatures (usually above 30 or at 37°C) is low (e.g. di Menna, 1955; Mok et al., 1984; Sylvester et al., 2015). Clinically relevant yeasts are not common or abundant in soils and they are probably introduced with animal feces and waste.

## 6 DISTRIBUTION OF SOIL YEASTS

The recent review by Botha (2011) describes the diversity of interactions of soil yeasts with the environment, including both abiotic and biotic factors. Soil yeasts respond to changes in abiotic factors, including soil organic matter content, pH, conductivity, temperature and availability of water and macronutrients, such as N, P, K, Na and Mg (e.g. Botha, 2006, 2011; Chernov, 2005; Sláviková & Vadkertiová, 2003; Vishniac, 2006a). Similarly, changes in the yeast community of soils correlate with soil moisture (or rainfall) following seasonal changes in forest soils (Sláviková & Vadkertiová, 2000), microclimate (Yurkov, Röhl, et al., 2016; Yurkov, Wehde, et al., 2016) and latitudinal changes of physico‐chemical environmental conditions (Chernov, 2005; Vishniac, 2006a). At the same time, abiotic soil parameters have little effect on soil yeast communities within the same type of habitat. It has been shown that yeast quantity, diversity and community structure reflect vegetation properties, such as age and management history, but not basic abiotic properties, including pH, nitrogen content and C/N ratio (Birkhofer et al., 2012; Yurkov et al., 2012). Likewise, yeast communities in Mediterranean forest soils reflected the properties of the forest cover, which in turn is shaped by the local precipitation regime (Yurkov, Wehde, et al., 2016).

The diversity and composition of soil yeast communities is influenced by vegetation, i.e. plant diversity and composition. Ascomycetous yeasts are more prominent in grassland and agricultural soils and the proportion of these yeasts increases with the intensity of land management (Sláviková & Vadkertiová, 2003; Yurkov et al., 2012). Agricultural practice is often associated with monoculture cropping, which negatively affects soil yeasts (reviewed in Vadkertiová et al., 2017). Orchard and vineyard soils often contain large numbers of ascomycetous yeasts and some of them (e.g. genera *Hanseniaspora*, *Metschnikowia* and *Ogataea*) can be also isolated from fruits (e.g. Kachalkin, Abdullabekova, Magomedova, Magomedov, & Chernov, 2015; Lachance, 2016; Sipiczki, 2016; Vadkertiová, Molnárová, Vránová, & Sláviková, 2012). Invasive plant species, which are not native to a specific location, often tend to spread, suppressing indigenous flora and causing damage to the environment. It has been recently demonstrated that soil yeast communities under invasive plants are also different from those under rural vegetation (Glushakova, Kachalkin, & Chernov, 2015a, 2016; Glushakova et al., 2015b). Compared with typical meadow vegetation, the abundances of *Saitozyma podzolica*, *Schwanniomyces castelli* and *Torulaspora delbrueckii* were negatively affected by the invasion of Impatiens parviflora, whereas the soil‐borne species *Apiotrichum dulcitum* and *Apiotrichum laibachii* were more prominent as a result of the invasion (Glushakova et al., 2015a). Similarly, *Candida vartiovaarae*, *Schwanniomyces castelli* and *Tausonia pullulans* were less abundant in a ruderal and invasive *Heracleum sosnowskyi* and *Aster salignus* (Glushakova et al., 2015b, 2016) regime. A common feature of all three studied floral invasions is an increased species richness trend and the proportion of ascomycetous yeasts, most of which are not typical for meadow soils. This observation is consistent with the earlier report of ascomycetous yeasts dominating soil yeast communities in managed grasslands (Yurkov et al., 2012).

Many yeast species are adapted to soil habitats. Some of them are widespread and others are found in a certain type of soil. Several studies that surveyed yeasts in a broad range of soils attempted to correlate soil properties with distribution of yeast taxa (e.g. Babjeva & Golovleva, 1969; Babjeva & Chernov, 1995; Chernov, 2005; di Menna, 1965a; Vishniac, 2006a). Chernov (2005) and Vishniac (2006a) performed the two largest studies of soil yeasts along a latitudinal gradient in the former USSR and western North America, respectively. They examined basic environmental parameters as factors that may influence the distribution of yeasts in these soils. Both authors reported substantial dissimilarity between sampling regions. In samples collected on the East European Plain, the quantity of yeasts showed a unimodal distribution reaching the highest values in boreal and temperate climates and rapidly declined towards the North and the South (Chernov, 2005). Similarly, the diversity of yeast communities increased from subtropical deserts to the tundra but most of the increase was observed in boreal climate between forest biotopes and the tundra (Chernov, 2005, 2013). Among potential indicator species figured *Saitozyma podzolica*, associated with acid well‐drained soils and *Nadsonia* (*Schizoblastosporion*) *starkeyi‐henricii*, frequent in cold and temperate hydromorphic soils (see also Babjeva & Blagodatskaya, 1972; di Menna, 1965b; Yurkov et al., 2012). Species of the genus *Naganishia* (*Cryptococcus* spp. in the Albidus clade, Filobasidiales, Tremellomycetes) dominated in desert soils (Chernov, 2005; Vishniac, 2006a). Cold soils, both polar and alpine, are inhabited by *Goffeauzyma gilvescens* (reviewed by Babjeva & Chernov, 1995; Connell et al., 2014; Zalar & Gunde‐Cimerman, 2014). A multivariate statistical analysis performed by Vishniac (2006a) showed that the two species *Naganishia albida* and *Filobasidium chernovii* (formerly *Cryptococcus* spp.) responded to elevated temperatures. The yeasts *Tausonia pullulans* and *Solicoccozyma terricola* were characteristic for temperate climates and *Solicoccozyma aeria* for arid climates in the analysis presented by Chernov (2005). Although soil yeasts respond to environmental parameters, mechanisms explaining their distribution patterns are not yet understood. The observed spatial heterogeneity and endemism of soil yeasts may result from undersampling or reflect the distribution and availability of ecological niches yeasts occupy in soils. In contrast to the common view on yeasts as free‐living soil organisms, their distribution may not depend on abiotic factors (e.g. Birkhofer et al., 2012) but is determined by plant, insect and fungal hosts and vectors.
